# Bone Marrow Mesenchymal Stem Cell-Derived Exosomes Inhibit Triple-Negative Breast Cancer Cell Stemness and Metastasis via an ALKBH5-Dependent Mechanism

**DOI:** 10.3390/cancers14246059

**Published:** 2022-12-09

**Authors:** Yun Hu, Hanyuan Liu, Xiudi Xiao, Qiao Yu, Rong Deng, Lixin Hua, Jinhua Wang, Xinwei Wang

**Affiliations:** 1 Department of General Surgery, The Affiliated Cancer Hospital of Nanjing Medical University & Jiangsu Cancer Hospital & Jiangsu Institute of Cancer Research, Nanjing 210009, China; 2 Department of General Surgery, Nanjing First Hospital, Nanjing Medical University, Nanjing 210012, China; 3 Department of Breast Surgery, Affiliated People’s Hospital of Jiangsu University & Zhenjiang First People’s Hospital, Zhenjiang 212000, China; 4 Department of Urology, The First Affiliated Hospital of Nanjing Medical University, Nanjing 210029, China; 5 Department of Female Tumor, The Affiliated Cancer Hospital of Nanjing Medical University & Jiangsu Cancer Hospital & Jiangsu Institute of Cancer Research, Nanjing 210009, China; 6 Department of Oncology, The Affiliated Cancer Hospital of Nanjing Medical University & Jiangsu Cancer Hospital & Jiangsu Institute of Cancer Research, Nanjing 210009, China

**Keywords:** triple-negative breast cancer, bone marrow mesenchymal stem cells, exosomes, m6A demethylase, ALKBH5, UBE2C, p53, stemness

## Abstract

**Simple Summary:**

Triple-negative breast cancer (TNBC) is a type of breast cancer characterized by a lack of hormone receptors expression and HER2 gene amplification, which presents with a high probability of metastasis. Bone marrow mesenchymal stem cell-derived exosomes have an influence in the development of various tumors. This study set out to analyze the interaction of the ALKBH5/UBE2C/p53 axis and the molecular mechanism mediated by BMSC-Exos in the stemness property of TNBC cells based on the data of The Cancer Genome Atlas and Gene Expression Omnibus as well as in vivo animal experiments. which renders varieties of potential molecular targets in cancer therapy of TNBC.

**Abstract:**

Background: Abnormal N6-methyladenosine (m6A) modification caused by m6A regulators is a common characteristic in various tumors. However, little is known about the role of m6A regulator AlkB homolog 5 (ALKBH5) in triple-negative breast cancer (TNBC). In this study, we analyzed the influence of ALKBH5 on the stemness of TNBC and the molecular mechanism using bioinformatics analysis and in vivo animal experiments. Methods: RNA expression data and single-cell RNA sequencing (scRNA-seq) data were downloaded from the TCGA and GEO databases. Following intersection analysis, key genes involved in the TNBC cell stemness were determined, which was followed by functional enrichment analysis, PPI and survival analysis. Exosomes were extracted from bone marrow mesenchymal stem cells (BMSC-Exos) where ALKBH5 inhibition assay was conducted to verify their function in the biological characteristics of TNBC cells. Results: Bioinformatics analysis revealed 45 key genes of ALKBH5 regulating TNBC cell stemness. In addition, UBE2C was predicted as a key downstream gene and p53 was predicted as a downstream signaling of ALKBH5. In vivo data confirmed that ALKBH5 upregulated UBE2C expression by regulating the m6A modification of UBE2C and reduced p53 expression, thus promoting the stemness, growth and metastasis of TNBC cells. BMSC-Exos suppressed the tumor stemness, growth and metastasis of TNBC cells and ALKBH5 shRNA-loaded BMSC-Exos showed a more significant suppressive role. Conclusion: Taken together, our findings indicated that ALKBH5 shRNA-loaded BMSC-Exos reduced TNBC cell stemness, growth and metastasis and define a promising strategy to treat TNBC.

## 1. Introduction

Triple-negative breast cancer (TNBC) is a type of breast cancer characterized by a lack of hormone receptors expression and HER2 gene amplification, which presents with a high probability of metastasis [1,2,3]. Cancer stemness, namely the stem cell-like phenotype in tumors, is responsible for the development of breast cancer metastasis [4]. Identifying the mechanism underlying TNBC stemness can enable the development of new therapeutic strategies for the prevention and treatment of TNBC.

Increasing studies have suggested the inhibiting property of bone marrow mesenchymal stem cell-derived exosomes (BMSC-Exos) in the development of various tumors [5,6]. BMSC-Exos can restrain the growth and progression of breast cancer cells in vitro and in vivo [7]. Exos can load proteins, lipids, nucleic acids, and methyladenosine RNA demethylase [8,9]. N6-methyladenosine (m6A) is the most abundant internal modification implicated in tumorigenesis; the m6A demethylase, a-ketoglutarate-dependent dioxygenase AlkB homolog 5 (ALKBH5), has demonstrated the promoting function in the development of breast cancer [10]. Reducing m6A expression by the knockdown of ALKBH5 is capable of inhibiting the viability, colony formation and cell migration of breast cancer cells, thus arresting the tumorigenesis of breast cancer [11]. Forced ALKBH5 expression can stabilize ubiquitin-conjugating enzyme E2C (UBE2C) epi-transcriptionally via the maintenance of lower m6A levels within its mature RNAs in non-small cell lung cancer [12].

Of note, UBE2C, an E2 ubiquitin-conjugating enzyme, has been reported to promote the progression of various cancers, including breast cancer [13]. UBE2C overexpression is associated with estrogen-dependent/independent proliferation in early hormone receptor-positive/HER2-negative breast cancer [14]. In addition, a previous study has highlighted the prognostic significance of UBE2C mRNA expression in the breast cancer patients since high UBE2C mRNA expression functions as an independent adverse prognostic factor for relapse and death [15]. The upregulation of UBE2C expression can enhance the ubiquitination and degradation of p53 [16]. Furthermore, p53 is a member of the p53 gene family involved in the development, differentiation and response to cellular stress, which is essential for cancer prevention [17]. A recent work has identified the ability of p53 overexpression to reduce the number of breast cancer cells with stemness property [18]. However, the role of p53 potential upstream regulators of ALKBH5 and UBE2C in the stemness of TNBC cells is still unclear. 

This study set out to analyze the interaction of the ALKBH5/UBE2C/p53 axis and the molecular mechanism mediated by BMSC-Exos in the stemness property of TNBC cells based on the data of The Cancer Genome Atlas (TCGA) and Gene Expression Omnibus (GEO) as well as in vivo animal experiments.

## 2. Materials and Methods

### 2.1. Data Retrieval and Analysis

RNA expression data (HTSeq-FPKM) and corresponding clinical information of 113 normal samples and 1089 TNBC (TCGA-BRCA) samples were downloaded from the TCGA database. The single-cell RNA sequencing (scRNA-seq) dataset GSE161529 was retrieved from the GEO database, including tumor tissue samples from 4 TNBC patients (22,322 cells in total). 

### 2.2. Weighted Gene Co-Expression Network Analysis (WGCNA)

WGCNA is a systems biology method used to describe gene association patterns between different samples. It can be applied for finding gene modules that change highly synergistically and identifying candidate biomarkers or therapeutic targets according to the interconnectivity of gene modules and the association between gene modules and phenotypes [19]. The identified highly co-expressed gene modules can be regarded as a set of genes specifically expressed by certain types of cells in the tumor tissue. 

Among the tumor tissue samples from 1089 TNBC patients, 10 outlier samples were removed, and the remaining 1079 samples were used for subsequent analysis. Grouping was conducted according to ALKBH5 high/low expression as well as patient survival, which was followed by network construction with the differential genes between groups as the input gene using the R “WGCNA” software package. An appropriate soft threshold was selected using the “pickSoftThreshold” function, and the adjacency matrix was transformed. A topological overlap matrix (TOM) was further calculated, and a hierarchical cluster dendrogram was constructed to divide similar gene expression into different modules with the minimum number of genes of 100 per module. The expression profile of each module was summarized based on the characteristic gene, and the correlation between characteristic genes and ALKBH5 expression and the survival of patients was calculated, with the most relevant modules selected for further analysis. 

### 2.3. Analysis of scRNA-seq Data

Low-quality cells were filtered after the expression matrix was obtained using 10× Genomics official analysis software Cell Ranger. By filtering single cells expressing less than 500 genes and cells with mitochondrial genes accounting for more than 25%, UMI and Gene correlation analysis was performed to infer the data quality. Next, dimensionality reduction in the highly variable gene expression matrix was conducted using Principal Component Analysis (PCA) in the R “Seurat” package, whereupon the JackStrawPlot and ElbowPlot functions were used to determine which principal components were selected for downstream analysis. The clustering results following the graph-based clustering algorithm were subjected to two-dimensional visualization with the UMAP algorithm. Cell clusters were annotated using the R “SingleR” package. Thereafter, trajectory analysis was performed using the R “Monocle2” software package. The differentially expressed genes in clustering results were first screened, which was followed by dimensionality reduction using DDRTree. Cells were then sorted and performed trajectory construction. A heat map of the expression of the sorted genes following clustering analysis was plotted to present the top 100 driver genes and the expression trend of the gene of interest in each cell cluster. 

### 2.4. Key Gene Acquisition

The jvenn tool was used to intersect the order gene and tumor stem cell marker gene associated with cell differentiation following trajectory analysis in scRNA-seq analysis with the module genes most related to ALKBH5 expression and the survival of TNBC patients in WGCNA. A Venn map was drawn to obtain the key genes.

### 2.5. Functional Enrichment Analysis

GO and KEGG enrichment analysis of the 45 key genes was performed using the R “clusterProfiler” package, with *p* < 0.05 as the threshold. GO enrichment analysis comprises a biological process (BP), cellular component (CC) and molecular function (MF). The top 20 entry identifiers of the three parts were selected to draw a bubble diagram. KEGG enrichment analysis was performed with *p* < 0.05 as the threshold.

### 2.6. Protein–Protein Interaction (PPI) Analysis and Survival Analysis

The proteins encoded by the key genes were imported into the STRING database, with “human” as species to construct a PPI network. The Degree value (the number of genes interacting with other genes in the PPI network; that is the core degree) was then calculated, and the top 15 genes were selected according to the Degree value for display. Thereafter, the correlation of 15 key genes and the survival of patients was analyzed using the GEPIA database.

### 2.7. Orthotopically Transplanted Tumor of TNBC in Mice and Metastasis Experiments

Female NOD/SCID immunodeficient mice (4–5 weeks; 18–22 g; Hunan SJA Laboratory Animal Co., Ltd., Hunan, China) were housed individually in the SPF laboratory at 25–27 °C and 45–50% humidity under a 12 h light/dark cycle. The mice were deprived of food 12 h before administration, with ad libitum access to food and water at other times. The mice were acclimated for one week before experiment. Animal experiments were approved by the Experimental Animal Ethics Committee of our hospital.

The TNBC cell line MDA-MB-231 was cultured in DMEM supplemented with 10% FBS, 1% antibiotic/antimycotic solution (15240096, Gibco, Grand Island, NY, USA), and 10 mg/mL gentamicin. The MDA-MB-231 cell suspension (5 × 10^6^ cells/0.2 mL) harboring oe-NC + sh-NC, oe-ALKBH5 + sh-NC and oe-ALKBH5 + sh-UBE2C, or 20 μg BMSC-Exos (suspended in 40 μL PBS; including Exos-sh-NC and Exos-sh-ALKBH5) was in situ injected into the mice, which was followed by the in situ injection of equal volumes of PBS. At 6 weeks after injection, the mice were euthanized. Tumor tissues were dissected and stored at −80 °C for subsequent analysis. 

293T cells were transfected with 3 μg pLenti-shNC or pLenti-sh-ALKBH5, 1 μg pCMV-VSV-G and 3 μg pCMV-Delta8.9 using Lipofectamine 3000 reagent (L3000001, Invitrogen, Carlsbad, CA, USA). Three shRNA sequences were designed and provided for qRT-PCR screening for optimal shRNA sequences. sh-UBE2C sequence: AGUGGUCUGCCCUGUAUGAdTdT. sh-ALKBH5 sequence: CUGAGAACUACUGGCGCAA. At 20 h after transfection, the medium was replaced with 12 mL of medium containing 5% FBS (10099141, Invitrogen). After approximately 48 h, the virus-containing supernatant was collected, filtered with a 0.45 μm cellulose acetate filter (SAMP2HNNB, Merck Millipore, Billerica, MA, USA), and stored at −80 °C for construction of the sh-ALKBH5 and sh-UBE2C cell lines (MDA-MB-231 cells and BMSCs). The virus-containing supernatant was diluted 3 folds with serum-free DMEM containing 10 μg/mL polycoagulamine (40804ES76, Yeasen, Shanghai, China). Cells with 50% confluence were cultured with virus-containing medium for 8 h and then with DMEM (10567022, Invitrogen) containing 10% FBS for 24 h. Next, 10 μg/mL of puromycin (540411, Sigma-Aldrich Trading Co., Ltd., Shanghai, China) was added to the medium to obtain stable cell lines.

Lentivirus-transduced MDA-MB-231 cells or BMSC-Exos were injected in situ into the mouse groin fat pad. After one week, the width (W) and length (L) of the tumors were measured using a vernier caliper once a week, and the tumor volume was calculated using the following formula: tumor volume = (W^2^ × L)/2. After 7 weeks, mice were euthanized after which tumor tissues were excised and the tumor was weighed and photographed with a camera. For the lung metastasis model, MDA-MB-231 cells or BMSC-Exos were injected into the mice via the tail vein. Mice were euthanized at 6 weeks after the injection. Lung tissues were removed, and the metastatic nodules were counted using a microscope. The presence of micro-metastases in the lung tissue was examined by HE staining.

### 2.8. BMSC Culture

Human BMSCs purchased from ATCC (Manassas, VA, USA) were cultured in serum-free DMEM for 2 days and centrifuged at 500× *g* for 10 min and at 2000× *g* for 30 min to remove dead cells and debris. The resulting supernatant was centrifuged at 100,000× *g* and 4 °C at high speed for 1 h, resuspended in PBS, and ultra-centrifuged under the same conditions. The pellet was obtained and stored at −80 °C.

### 2.9. Isolation of Exos

Serum-containing medium was ultra-centrifuged overnight at 100,000× *g* and 4 °C to remove Exos from the serum. BMSCs were cultured in conditioned medium (CM; DMEM-F12 + 10% Exo-free serum) for 48 h, and the culture supernatant was harvested for Exo isolation. The supernatant was centrifuged at 500× *g* and 4 °C for 10 min, then at 2000× *g* for 30 min to remove cell debris and at 10,000× *g* and 4 °C for 20 min to remove large vesicles. Following filtration through a 0.22 μm filter, the supernatant was centrifuged at 110,000× *g* for 70 min. The pellet was then resuspended in PBS, ultra-centrifuged at the same conditions, resuspended in 100 μL sterile PBS and frozen at −20 °C.

### 2.10. Transmission Electron Microscope (TEM)

Exo resuspension was ultra-centrifuged, fixed with fixative (2% paraformaldehyde and 2.5% glutaraldehyde) for 1 h, PBS (15 min / time) at 4 °C, and then with 1% osmium acid for 1.5 h. Next, the resuspension was dehydrated with gradient alcohol, immersed in epoxy resin overnight, embedded and polymerized at 35 °C, 45 °C and 60 °C for 24 h, followed by ultrathin sectioning. After staining, the sample was observed under a TEM (JEM-1010, JEOL, Tokyo, Japan). 

### 2.11. Nanoparticle Tracking Analysis (NTA)

Exo resuspension was diluted, an appropriate amount of which was detected with a nanoparticle tracking analyzer (Malvern Instruments, Malvern, UK). The diluted samples with the concentration fluctuated at 1 × 10^8^–9 × 10^8^ cells/mL were collected for detection. The operation software with appropriate background gray scale was used, the movement trajectory of the particles was recorded, and the diluted sample concentration and size distribution map were output. The Exo concentration of the stock solution was calculated according to the dilution multiple. 

### 2.12. Western Blot

Total protein from the cells was extracted, separated with 10% SDS-PAGE and transferred onto PVDF membranes. The membrane was blocked with 5% skimmed milk for 1 h at room temperature and probed with the diluted rabbit polyclonal primary antibodies (Abcam) against TSG101 (ab30871, 1:2000), CD63 (ab216130, 1:1000), calnexin (ab75801, 1:2000), ALKBH5 (ab195377, 1:1000), and GAPDH (ab8245, 1:10,000) at 4 °C overnight and with HRP-labeled secondary antibody IgG (1:1000) for 1 h. ECL reagent (SW2010, Beijing Solarbio Science & Technology Co., Ltd., Beijing, China) was used to visualize the results by the IS gel image analysis system. Band intensities were quantified using ImageJ software.

### 2.13. qRT-PCR

Total RNA was extracted from cells using TRIzol reagent (16096020, Thermo Fisher Scientific Inc., Waltham, MA, USA), the concentration and purity of which were determined using a NanoDrop One/OneC microvolume spectrophotometer (A260/A280 = 2.0, concentration greater than 5 μg/μL). For mRNA detection, a cDNA first-strand synthesis kit (D7168L, Shanghai Beyotime Biotechnology Co., Ltd., Shanghai, China) was used to synthesize cDNA. qRT-PCR was conducted using a qRT-PCR kit (Q511-02, NanJing Vazyme Biotech Co., Ltd., Nanjing, China). GAPDH was regarded as an internal reference, and the fold changes were calculated by relative quantification (2^−ΔΔCt^ method). The primer sequence was designed and provided by Shanghai Sangon Biotechnology Co. Ltd. (Shanghai, China), as shown in Supplementary Table S1. 

### 2.14. Immunohistochemistry

Paraffin tissue samples were sectioned at the thickness of 5 μm, dehydrated, treated with 3% hydrogen peroxide at room temperature for 10 min and blocked with normal goat serum for 10 min. Next, the sections were immunostained with rabbit antibodies (Abcam) against ALKBH5 (1:2000, ab195377), UBE2C (1:500, ab252940), p53 (1:100, ab32389), Ki67 (1:200, ab15580), NANOG (1:200, ab109250), OCT4 (1:250, ab200834), and SOX2 (1:100, ab92494) at 4 °C overnight. The next day, the sections were incubated with biotin-labeled goat anti-rabbit secondary antibody (1:200, BA1003, Boster Biological Technology Co. Ltd., Wuhan, Hubei, China) at 37 °C for 20 min and then with 50 μL streptavidin biotin peroxidase complex at room temperature for 10 min. The sections were exposed to DAB substrate, which was followed by hematoxylin counterstaining and dehydration treatment. The staining images were obtained using a microscope. The normal positive cells were brownish yellow, and the positive staining was statistically analyzed by ImageJ software.

### 2.15. HE Staining

Sections were dewaxed, hydrated with gradient alcohol and stained with hematoxylin (G1140, Solarbio, Beijing，China) for 2 min. Next, the sections were hydrolyzed with 1% hydrochloric acid ethanol for 10 s, counterstained with eosin for 1 min, dehydrated in ascending series of alcohol, cleared in xylene and mounted with neutral gum. Finally, the sections were observed under an optical microscope (XP-330, Shanghai Bingyu Optical Instrument Co., Ltd., Shanghai, China). 

### 2.16. Bioinformatics Analysis Procedures and In Vivo Verification

The general flow of the present study was as follows: (1) TCGA-BRCA data were downloaded from the TCGA database, and we performed WGCNA; patients were grouped according to ALKBH5 expression as well as patient survival; module genes most related to ALKBH5 expression and the survival of TNBC patients were screened; (2) the scRNA-seq dataset GSE161529 was retrieved from the GEO database, which was followed by data quality control, filtering, PCA, UMAP cluster analysis, cell annotation, and trajectory analysis; the order gene and tumor stem cell marker gene associated with cell differentiation following trajectory analysis were identified; (3) the module gene, order gene, and marker gene were intersected, and 45 key genes of ALKBH5 regulating tumor cell stemness were determined; (4) GO and KEGG enrichment analysis of the 45 key genes was conducted to clarify the key downstream pathways (p53 pathway); (5) the PPI network of proteins encoded by the 45 key genes was constructed and in combination with the survival analysis, a core gene (UBE2C) was obtained; (6) an orthotopic transplantation model of TNBC and a metastasis model in mice were developed to elucidate the effect of ALKBH5 on tumor growth and metastasis via regulation of the UBE2C/p53 axis and clarify the application value of BMSC-Exos in TNBC intervention.

### 2.17. Statistical Analysis

Statistical comparison was performed using an unpaired *t*-test when only two groups were compared or by one-way analysis of variance (ANOVA) when more than two groups were compared. Variables were analyzed at different time points using repeated measures ANOVA. All statistical analyses were completed with SPSS 22.0 software (IBM, Armonk, NY, USA) and GraphPad Prism 8.0, with *p* < 0.05 as a level of statistical significance. Measurement data were expressed as mean ± standard deviation. 

## 3. Results

### 3.1. Identification of Target Genes of ALKBH5 in TNBC

**Figure 1:**
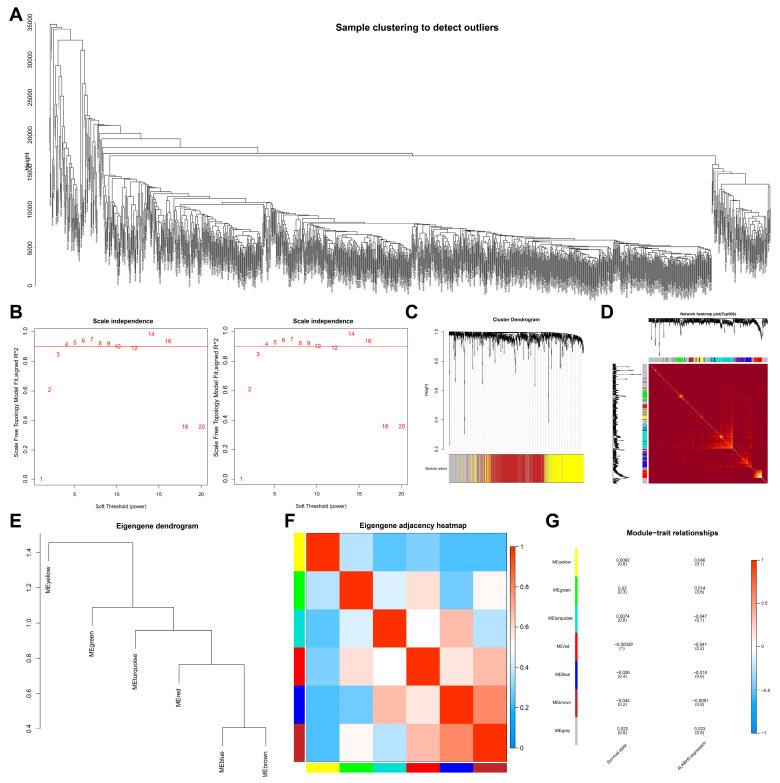
Screening of module genes associated with ALKBH5 expression by WGCNA. (**A**): A cluster diagram of TCGA-BRCA samples (*n* = 1,089). One “branch” represents one sample; (**B**): The scale-free fitting index of network topology using soft threshold power analysis. Soft threshold (power) in the left represents the weight, and the ordinate represents the correlation between connectivity k and p (k). It is generally required that power with the correlation reaching 0.8 is used as a *β* value. Soft threshold (power) in the right indicates weight, and the ordinate represents the average connectivity degree; (**C**): A gene co-expression network constructed by WGCNA. Each color represents a module in the network; (**D**): Correlation analysis of the co-expressed gene interaction. Different colors of the abscissa and ordinate represent different modules, and the brightness of yellow in the middle indicates the connection degree of different modules. No significant difference in the interaction between modules indicates that the scale has a high degree of independence between the modules; (**E**): Cluster analysis of different modules; (**F**): Correlation analysis of different modules. Color from blue to red indicates the overall expression of the first principal component gene module eigengene from low to high; (**G**): Correlation analysis of the different modules with the ALKBH5 expression. Different colors indicate the different modules.

To screen downstream targets of ALKBH5, we constructed a scale-free co-expression network to identify the module genes most related to ALKBH5 expression and the survival of TNBC patients using the R “WGCNA” package. A total of 16,381 genes were annotated in the TCGA-BRCA dataset. The standard deviation for each gene expression was calculated, and the first 15% of 9010 genes according to the standard deviation order were taken for subsequent WGCNA analysis. As shown in Figure 1A, 10 outlier samples were found and then deleted, with 1079 samples remaining. Grouping was conducted according to ALKBH5 high/low expression as well as patient survival. The threshold of soft-power *β* = 4 was set to build a co-expression network (Figure 1B), where the minModules size was set to 100, deepSplit was set to the default value of 2, and cut height was set to 0.25. The gene was finally divided into seven modules, which were each given a different color (Figure 1C).

Correlation between the seven modules was analyzed, and a heat map was drawn to indicate the relatively independent gene expression of each module (Figure 1D). The eigengenes values of the seven modules were calculated and clustered based on their correlation to obtain the expression similarity between the individual module, where the gray modules were outliers and not included (Figure 1E,F). In addition, the brown module was most associated with ALKBH5 expression and the survival of patients (Figure 1G). The above results showed that 601 brown module genes in breast cancer may be the most related genes with ALKBH5 expression and thus were used for subsequent analysis.

### 3.2. Quality Control, Filtering, and PCA of scRNA-seq Data from Human TNBC Tissue Samples

**Figure 2:**
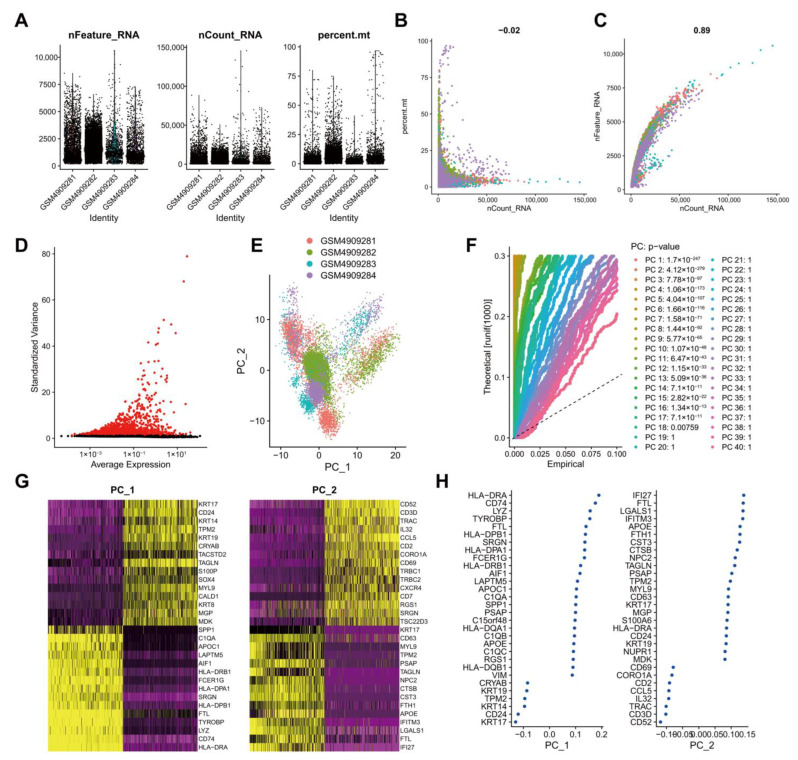
Analysis of the scRNA-seq data from human TNBC tissue samples. (**A**): Expression of scRNA-seq data. Each dot represents one cell, nFeature_RNA ordinate represents the number of genes expressed in each cell, nCount_RNA ordinate represents the number of UMI in each cell, and percent.mt ordinate indicates the proportion of the UMI of the mitochondrial genes per cell to the total UMI of each cell; (**B**): A scatter plot of the relationship between the UMI and the total number of genes; (**C**): Highly variable genes in tumor tissue samples following variance analysis. Red dots represent highly variable genes, and black dots represent invariable genes; (**D**): PCA of cell cluster; (**E**): *p* values of each principal component compared using the JackStrawPlot function. Above the dotted line indicates a *p* value less than 0.05; (**F**): Principal components used for the subsequent analysis determined by the ElbowPlot function (the turning point was determined according to the variance change); (**G**): A dot plot of the related genes in the top two principal components. The larger absolute value of the abscissa value reflects the higher correlation; (**H**): A heat map of the expression of related genes in the top two principal components. The darker color reflects the greater expression.

To screen genes with significant changes in TNBC tissues at the cellular level, we retrieved the scRNA-seq dataset GSE161529 containing breast cancer patient tumor tissue samples from the GEO database. Low-quality cells were filtered after the expression matrix was obtained using Cell Ranger software. By filtering single cells expressing less than 500 genes and cells with mitochondrial genes accounting for more than 25%, 20,282 cells were obtained from 22,322 cells (Figure 2A). Data quality analysis data using UMI and Gene correlation analysis showed that the correlation coefficient between nCount and percent.mt was r = −0.02, and that between nCount and nFeature was r = 0.89, which indicated the good cell quality (Figure 2B). After the standardization of each cell, in order to better utilize PCA for dimensionality reduction in cells, it is necessary to screen highly variable genes in the entire dataset for subsequent analysis. By calculating the mean and variance of each gene, the top 2000 genes with high variation were screened for subsequent analysis (Figure 2C). These results suggested a large number of highly variable genes in the tumor tissue samples of breast cancer patients.

For multi-dimensional evaluation of our samples, we applied the RunPCA function in Seurat software package for PCA, and the results were visualized. After the samples were normalized, there was no obvious batch effect among the samples (Figure 2D). To determine which principal components were selected for downstream analysis, we used the JackStrawPlot function to visualize the top 40 principal components and compared the distribution of *p* values of each principal component relative to the mean distribution. “Important” principal components usually have a very small *p* value (in the solid line above the dotted line) and can fully reflect the information contained in the previously selected highly variable genes. As illustrated in Figure 2E, *p* values of the top 18 principal components were less than 0.05. Following the ElbowPlot function, the results exhibited a significant turn at 10 points, so the top 10 principal components were selected for subsequent UMAP analysis (Figure 2F). Cells and genes were ranked based on PCA scores, and we selectively displayed the 30 major component genes in the top two principal components (Figure 2G) and plotted a heat map (Figure 2H). 

### 3.3. UMAP Cluster Analysis and Trajectory Analysis of scRNA-seq Data from Human TNBC Tissue Samples

**Figure 3:**
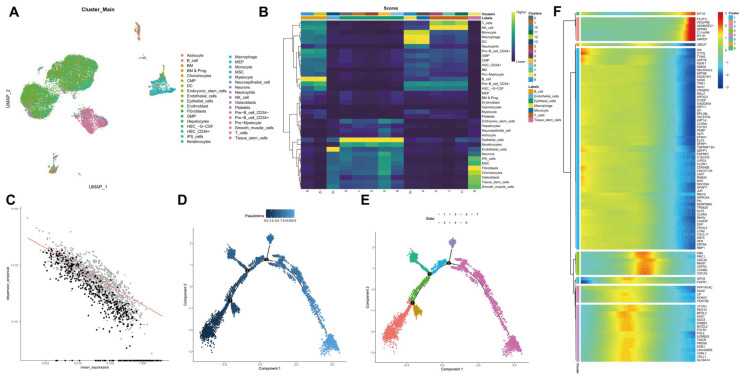
scRNA-seq data from human TNBC tissue samples are subjected to UMAP cluster analysis and trajectory analysis. (**A**): Cell types following UMAP cluster analysis; (**B**): Cell clusters are annotated into seven cell types using the R “SingleR” package; (**C**): Order gene visualization results; (**D**): Cell evolution trajectory display according to pseudotime. Color from dark to light indicates “time” proceeding; (**E**): Cell evolution trajectory display according to differentiation stage. Different colors represent different cell clusters; (**F**): A heat map showing the expression of the top 100 driver genes. The color change in the central indicates gene expression change.

We further performed the cluster analysis of cells based on the aforementioned top 10 principal components. The cluster results are shown in Figure 3A. In addition to common immune cells, fibroblasts, endothelial cells and tumor stem cells can be found. Cell cluster annotation results indicated that these clusters were annotated into seven major types of cells: B cells, endothelial cells, epithelial cells, macrophages, monocytes, T cells, and tissue stem cells (Figure 3B). 

Thereafter, trajectory analysis was performed using the R “Monocle2” software package, and the visualization results of trajectory construction genes (sequencing genes) are shown in Figure 3C. Data dimensionality reduction was performed using DDRTree, and cells were sorted according to the expression trend of order genes to complete the trajectory construction. Pseudotime is the probability calculated by the “Monocle2” software package based on cellular gene expression information, indicating the order of time, with the root on the left and the branch on the right (Figure 3D). At the same time, the display was followed according to state, and the cell evolution of the tumor microenvironment was divided into seven stages with three important branch nodes (Figure 3E). It can be seen that the states of each cell type are “active” in the tumor progression, which may be closely related to the key genes with dynamic changes. Therefore, we conducted cluster analysis of the order genes and then plotted a heat map showing their expression, with the top 100 driver genes shown (Figure 3F). The literature has reported that tumor cell dedifferentiation showing stemness may be an important mechanism for tumor recurrence and metastasis [20]. Therefore, we extracted 1027 marker genes and 465 order genes from the tissue stem cells cluster for subsequent analysis. 

### 3.4. ALKBH5 May Affect TNBC Growth and Metastasis through p53 Signaling and Cell Cycle-Related Pathways

**Figure 4:**
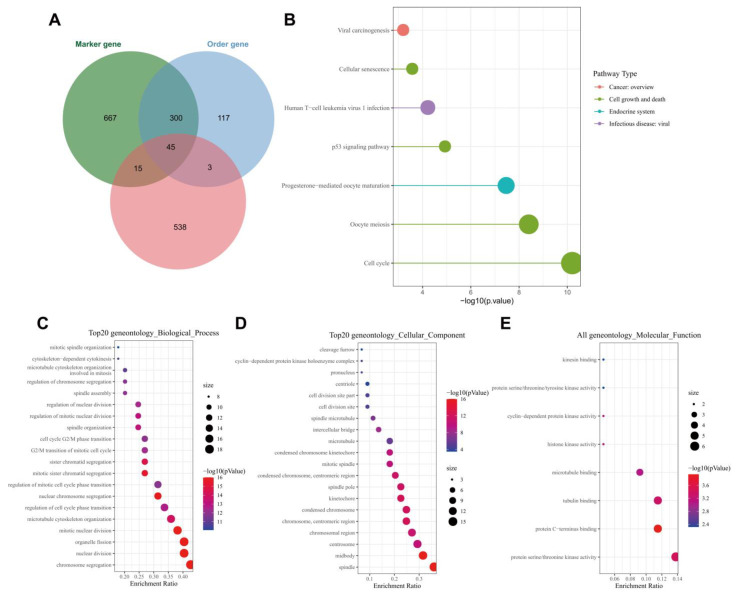
Functional enrichment analysis of the key genes. (**A**): Venn diagram of tumor stem cell-related marker genes, order gene and module genes; (**B**): KEGG enrichment analysis of the 45 key genes. The color indicates the pathway categories and the circle size represents the number of targets enriched; (**C**): A bubble diagram of the GO-BP analysis results of the 45 key genes; (**D**): A bubble diagram of the GO-CC analysis results of the 45 key genes; (**E**): A bubble diagram of GO-MF analysis results of the 45 key genes. In panels (**C**–**E**), the circle color indicates the *p* value, the circle size represents the number of targets enriched, and the abscissa represents the proportion of enriched targets and the ordinate indicates the pathway.

Following the Venn diagram analysis of 601 genes related to ALKBH5 expression following WGCNA, 1027 tumor stem cell-related marker genes and 465 order genes with alteration in tumor following scRNA-seq data analysis, a total of 45 key genes were identified (Figure 4A). KEGG analysis results showed that the 45 key genes were mainly enriched in the cell cycle and p53 signaling (Figure 4B). The ability of p53 to induce cell cycle arrest, DNA repair, senescence, and apoptosis has been largely reported; moreover, p53 also participates in embryogenesis and various differentiation programs, which can inhibit the dedifferentiation of mature somatic cells [21,22].

GO functional enrichment analysis results showed that the 45 key genes were predominantly enriched in the BP of chromosome segregation, mitotic nuclear division, nuclear division, organelle fission, nuclear chromosome segregation, CC of the midbody, spindle, chromosome, centromeric region, kinetochore and condensed chromosome and MF of protein C-terminus binding, protein serine, threonine kinase activity, tubulin binding, histone kinase activity and cyclin-dependent protein kinase activity (Figure 4C–E). These results were in line with those of KEGG analysis that genes were mainly enriched in cell cycle-related pathways. Meanwhile, published data have confirmed that tumor cells may re-enter the cell cycle and tend to dedifferentiate to maintain stemness, thus affecting tumor occurrence and progression [23]. These lines of evidence allowed us to propose that ALKBH5 may regulate breast cancer dedifferentiation, increase tumor cell stemness and promote TNBC growth and metastasis by mediating p53 signaling.

### 3.5. UBE2C May Be a Key Downstream Gene Involved in the ALKBH5 Function

**Figure 5:**
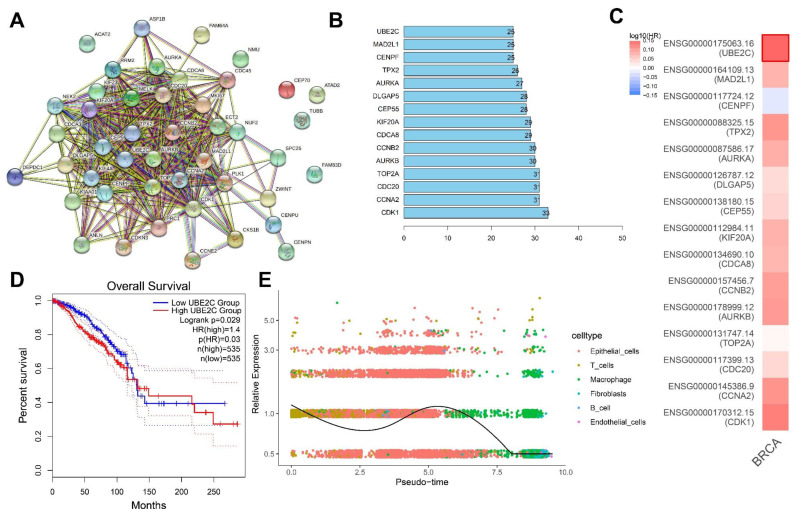
Screening of the core downstream genes of ALKBH5. (**A**): A PPI network of proteins encoded by the 45 key genes constructed using the STRING database. Each dot represents the protein encoded by the key gene and the connecting line indicates the existence of PPI; (**B**): The top 15 core genes determined based on the Degree values. The abscissa is the Degree value, namely the number of connected genes; (**C**): GEPIA2 analysis of the correlation of 15 core genes with the survival of TNBC patients. Red indicates the high-risk genes, blue indicates the low-risk genes, darker color indicates higher correlation, and bold border indicates *p* < 0.05; (**D**): Survival curve of correlation of the UBE2C expression with the survival of TNBC patients (n = 1070); (**E**): Dynamic expression of UBE2C following trajectory analysis of the scRNA-seq data. Dots with different colors indicate different cell types.

To further identify the key downstream genes of ALKBH5, we conducted a PPI network of proteins encoded by the 45 key genes using the STRING database (Figure 5A), following by ranking based on the Degree value, with the top 15 core genes displayed in Figure 5B. GEPIA2 analysis of the correlation of 15 core genes with the survival of TNBC patients indicated that UBE2C was most associated with the survival (Figure 5C). 

Further analysis found that TNBC patients with high UBE2C expression had a worse prognosis (Figure 5D). The results of the trajectory analysis of the scRNA-seq data showed that UBE2C expression was dynamically changed in each cell cluster (Figure 5E). Existing evidence has shown that the overexpression of UBE2C aggravates patient prognosis by promoting breast cancer cell proliferation [14]. The upregulation of ALKBH5 maintains lower m6A levels in UBE2C mature RNAs, which are post-transcriptionally stabilized to upregulate the UBE2C expression [12]. Meanwhile, UBE2C enhances the ubiquitination of p53 and promotes its degradation in endometrial cancer cells [24]. Therefore, UBE2C can play a role in TNBC development and, specifically, ALKBH5 may promote TNBC progression by regulating the m6A modification of UBE2C, upregulating UBE2C expression and blocking the p53 signaling.

### 3.6. ALKBH5 Enhances TNBC Cell Stemness and Promotes Tumor Growth and Metastasis via the UBE2C/p53 Axis in Mice

**Figure 6:**
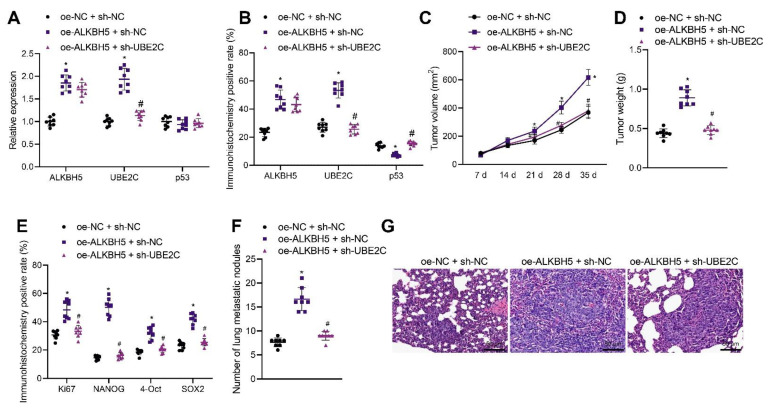
ALKBH5 accelerates TNBC cell growth and metastasis via the UBE2C/p53 axis in vivo. Mice were treated with oe-ALKBH5 along or in combination with sh-UBE2C. (**A**): mRNA expression of ALKBH5, UBE2C and p53 in the tumor tissues of mice determined by qRT-PCR; (**B**): Positive expression of ALKBH5, UBE2C, and p53 proteins in the tumor tissues of mice determined by immunohistochemistry; (**C**): Tumor volume of mice; (**D**): Tumor weight of mice; (**E**): Positive expression of Ki67, NANOG, OCT4, and SOX2 proteins in the tumor tissues of mice determined by immunohistochemistry; (**F**): Statistics of number of lung metastatic nodules in mice; (**G**): Lung metastasis of breast cancer cells analyzed by HE staining. n = 8. * *p* < 0.05, compared with the mice treated with oe-NC + sh-NC and # *p* < 0.05, compared to the mice treated with oe-ALKBH5 + sh-NC. Measurement data are expressed as mean ± standard deviation and comparisons between multiple groups were conducted by one-way ANOVA or repeated measures ANOVA. Oe-: overexpress; sh-: RNA-Short hairpin RNAs.

We moved to explore the effect of ALKBH5 regulating the UBE2C/p53 axis on the tumorigenesis and metastasis of TNBC cells in vivo. shRNA and an expression vector of ALKBH5 and UBE2C were applied to knock down and overexpress ALKBH5 and UBE2C, respectively. qRT-PCR and immunohistochemistry results revealed that the overexpression of ALKBH5 in the tumor tissues of mice increased the expression of ALKBH5 and UBE2C in both mRNA and protein level, whereas it decreased p53 only at the protein level. The silence of UBE2C partially rescues the effect of ALKBH5 on UBE2C and p53 (Figure 6A,B). These results suggest that ALKBH5 interacts with UBE2C and p53 and promotes UBE2C-induced ubiquitination of p53 in vivo.

The tumor volume and weight of oe-ALKBH5 + sh-NC-treated mice were increased but opposite results were noted in the presence of oe-ALKBH5 + sh-UBE2C (Figure 6C,D), suggesting that the tumor-promoting effect of ALKBH5 can be partially reversed by the silencing of UBE2C. The immunohistochemistry results demonstrated that the positive expression of Ki67, NANOG, OCT4, and SOX2 was increased in the tumor tissues of mice following ALKBH5 overexpression, the effect of which was reversed by the silencing of UBE2C (Figure 6E). As shown in Figure 6F, the number of lung metastatic nodules was increased following ALKBH5 overexpression, but it was decreased upon the silencing of UBE2C. HE staining further confirmed obvious cancer metastasis in the lung tissues of mice treated with oe-ALKBH5 + sh-NC, whereas the metastasis was decreased in the presence of oe-ALKBH5 + sh-UBE2C (Figure 6G). Overall, these results indicate that ALKBH5 can promote TNBC cell tumorigenesis and metastasis in vivo by upregulating the expression of UBE2C and reducing that of p53. 

### 3.7. ALKBH5 shRNA-Loaded BMSC-Exos Inhibits TNBC Cell Stemness and Retards Tumor Growth and Metastasis in Mice

BMSC-Exos were previously reported to inhibit the development of various tumors. Therefore, we determined whether BMSC-Exos could inhibit the progression of TNBC and whether ALKBH5 shRNA-loaded BMSC-Exos had better tumor-suppressive effects. Exos were isolated from BMSCs and identified. TEM results showed that Exos were round and showed oval membranous vesicles with a disc-like structure and a contact capsule and basically similar shape; in addition, the diameter of Exos ranged about 40–100 nm. Meanwhile, Western blot results found that Exo markers CD63 and TSG101 were positively expressed while the ER marker Calnexin was negatively expressed in the Exos (Supplementary Figure S2B). Moreover, NTA suggested that the main particle size of Exos was 50–200 nm, with the mean particle size of 115.1 nm, and the particle size with the highest concentration (8 × 10^7^ particle/mL) was 108.2 nm (Supplementary Figure S2C). The above results confirmed the successful extraction of BMSC-Exos.

**Figure 7:**
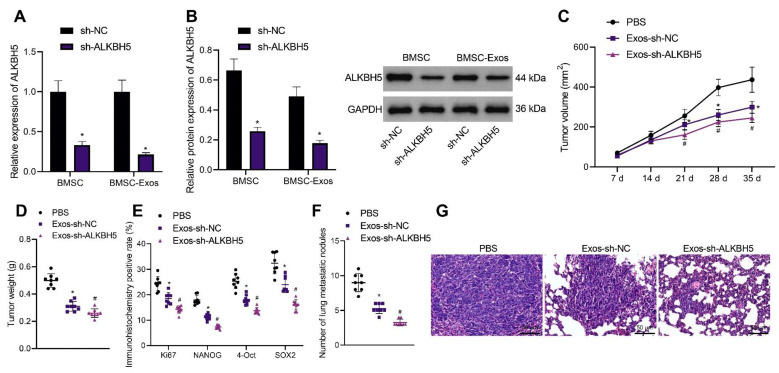
ALKBH5 shRNA-loaded BMSC-Exos reduces TNBC cell stemness and delays tumor growth and metastasis in vivo. (**A**): mRNA expression of ALKBH5 in the BMSCs and BMSC-Exos following ALKBH5 silencing determined by qRT-PCR; (**B**): The protein expression of ALKBH5 in BMSCs and BMSC-Exos following ALKBH5 silencing determined by Western blot; Mice were treated with ALKBH5 negative control-loaded BMSC-Exos (Exos- sh-NC) or ALKBH5 shRNA-loaded BMSC-Exos (Ex-os-sh-ALKBH5). (**C**): Tumor volume of mice; (**D**): Tumor weight of mice; (**E**): Positive expression of Ki67, NANOG, OCT4, and SOX2 proteins in the tumor tissues of mice determined by immunohistochemistry; (**F**): Statistics of number of lung metastatic nodules in mice; (**G**): Lung metastasis of breast cancer cells analyzed by HE staining. n = 8. * *p* < 0.05, compared with the mice treated with PBS and # *p* < 0.05, compared to the mice treated with Exos-sh-NC. Measurement data are expressed as mean ± standard deviation and comparisons between multiple groups were conducted by one-way ANOVA or repeated measures ANOVA. Statistical comparison was performed using unpaired t-test when only two groups were compared.

The results of qRT-PCR and Western blot showed a decrease in ALKBH5 expression in the BMSCs and BMSC-Exos following ALKBH5 silencing (Figure 7A,B). In addition, the tumor volume and weight of Exos-sh-NC- or Exos-sh-ALKBH5-treated mice were inhibited, with the latter showing a more obvious inhibition (Figure 7C,D). The positive expression of Ki67 and NANOG, OCT4, and SOX2 proteins was found to be decreased in the tumor tissues of mice treated with Exos-sh-NC, which was further reduced in the presence of Exos-sh-ALKBH5 (Figure 7E).

Moreover, treatment with Exos-sh-NC decreased the number of lung metastatic nodules, and a more prominent decrease was found upon treatment with Exos-sh-ALKBH5 (Figure 7F). HE staining results revealed that Exos-sh-NC improved the cancer metastasis in the lung tissues of mice, and Exos-sh-ALKBH5 led to more evident improvement (Figure 7G). Taken together, BMSC-Exos can reduce TNBC cell stemness and inhibit TNBC tumor growth and metastasis in vivo (Supplementary Figure S1). Notably, ALKBH5 shRNA-loaded BMSC-Exos exhibited more inhibitory effects on the tumor growth and metastasis.

## 4. Discussion

**Figure 8:**
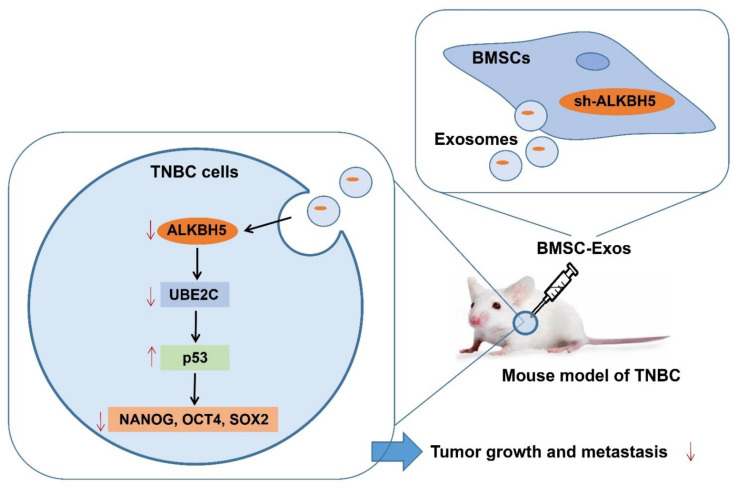
Schematic diagram of the mechanism by which BMSC-Exos affect the growth and metastasis of TNBC. ALKBH5 may promote TNBC cells to acquire stemness by mediating the UBE2C/p53 axis, thereby promoting TNBC growth and metastasis. ALKBH5 shRNA-loaded BMSC-Exos significantly inhibits the growth and metastasis of TNBC.

Based on bioinformatics analysis and in vivo animal experiments, this study unveiled the key molecular mechanisms by which BMSC-Exos mediating the ALKBH5/UBE2C/p53 axis affected the TNBC growth and metastasis. Specifically, ALKBH5 can induce the stemness of TNBC cells by upregulating UBE2C and downregulating p53, thereby promoting TNBC growth and metastasis. However, ALKBH5 shRNA-loaded BMSC-Exos inhibits the growth and metastasis of TNBC (Figure 8).

This study initially revealed that ALKBH5 may increase tumor cell stemness and promote TNBC growth and metastasis. Indeed, m6A regulators have been shown to play the most important role in TNBC [25]; ALKBH5 is remarkably upregulated in TNBC tumor tissues and acts as an independent unfavorable prognostic factor in this cancer [26]. Additionally, ALKBH5 decreases breast cancer sensitivity to doxorubicin by removing the m6A methylation of BRCA1 for mRNA stabilization and increases DNA repair competency [27]. Knockdown of ALKBH5 in MDA-MB-231 cells significantly weakens their capacity for tumor initiation due to the reduced breast cancer stem cells [28]. Notably, published data have identified the imperative role of m6A regulators in the stemness score of human cancers [29]. The possible role of ALKBH5 in the tumor cell stemness of TNBC warrants further investigations. 

Subsequent PPI and survival analysis results demonstrated that UBE2C may be a key downstream gene involved in the ALKBH5 promoting function in the TNBC cell stemness and tumor progression where ALKBH5 regulated the m6A modification of UBE2C and consequently upregulated UBE2C expression. Consistently, UBE2C has been found to be epi-transcriptionally stabilized with the maintenance of lower m6A levels within its mature RNAs in response to the increase in ALKBH5 expression in non-small cell lung cancer [12]. The UBE2C protein can be used as a prognostic marker in node-positive breast cancer due to its potential as an independent factor for metastasis-free survival and overall survival [30]. UBE2C is overexpressed in breast cancer tissues and functions as a cellular proto-oncogene to promote the proliferation of breast cancer cells [31]. Tongue squamous cell carcinoma cells with UBE2C silencing exhibit lowered expression of the cancer stemness markers ALDH1/A2, CD44, CD166 and EpCAM, indicating the role of UBE2C in controlling the cancer stemness [32]. Targeting the ALKBH5/UBE2C axis may create new opportunities for the prevention of TNBC cell stemness and the resultant tumorigenesis.

In addition, in vivo experimental results of the current study identified that mediation of the UBE2C/p53 axis was associated with the promoting role of ALKBH5 in the TNBC cell stemness and tumor growth and metastasis. UBE2C downregulation leads to an increase in the expression of p53 and UBE2C can facilitate p53 ubiquitination and degradation in endometrial cancer cells [24]. The loss of p53 facilitates cancer stemness and thus contributes to the progression of basal-like breast cancer [33]. The knockdown of p53 has been highlighted to accelerate the growth and metastasis of breast cancer cells [34]. A recent study has illustrated that p53 has binding sites in the ALKBH5 promoter and can induce the activation of ALKBH5 transcription, which acts as a feedback loop to regulate the m6A modification in pancreatic cancer [35]. This report results differ from ours in that p53 was a critical downstream pathway of ALKBH5, which may be the difference of the investigated subjects and perspective. 

The final results of this study demonstrated that ALKBH5 shRNA-loaded BMSC-Exos inhibited TNBC cell stemness and retarded tumor growth and metastasis in mice. LNA-antimiR-142-3p-loaded BMSC-Exos reduces the colony formation capability of breast cancer stem cells in vitro and reduces the tumorigenicity of breast cancer stem cells in vivo [36]. In addition, BMSC-derived exosomal miR-16-5p hinders the epithelial–mesenchymal transition of breast cancer cells and tumor growth in nude mice [7]. More importantly, MSC-Exos function to block the stemness of cancer stem cells of hepatocellular carcinoma [37]. BMSC-Exos also significantly reduces the tumor stemness of pancreatic cancer cells [38]. These lines of evidence support the present findings of BMSC-Exos inhibiting tumor stemness and the consequent growth and metastasis.

Collectively, our results reveal a novel mechanism by which ALKBH5 shRNA-loaded BMSC-Exos suppresses TNBC cell stemness and retards tumor growth and metastasis through the UBE2C/p53 axis, which renders varieties of potential molecular targets in cancer therapy of TNBC.

## 5. Conclusions

Our findings initially revealed that ALKBH5 may increase tumor cell stemness and promote TNBC growth and metastasis, and it indicated that ALKBH5 enhances TNBC cell stemness and promotes tumor growth and metastasis via the UBE2C/p53 axis. Targeting the ALKBH5/UBE2C axis may create new opportunities for the prevention of TNBC cell stemness and the resultant tumorigenesis. It renders varieties of potential molecular targets in the cancer therapy of TNBC. 

## Data Availability

All data that support the findings of this study are available from the corresponding authors upon reasonable request.

## Supplementary Materials

The following supporting information can be downloaded at: https://www.mdpi.com/article/10.3390/cancers14246059/s1, Figure S1: Uncropped Western blot figures for relevant result; Figure S2: Isolation and identification of the BMSC-Exos; Table S1: Primer sequences for qRT-PCR.
