# Supplementary material for: Bone Marrow Mesenchymal Stem Cell-Derived Exosomes Inhibit Triple-Negative Breast Cancer Cell Stemness and Metastasis via an ALKBH5-Dependent Mechanism
Source: Cancers (Basel). 2022 Dec 9;14(24):6059. doi: 10.3390/cancers14246059 (PMC9776833; doi:10.3390/cancers14246059)
Supplement: Supplementary file 1 [file cancers-14-06059-s001.zip › Table S1.pdf]

Supplementary Table S1. Primer sequences for qRT-PCR

Gene	Sequence
ALKBH5 (human)	Forward: 5′-GCTGGTTGCTCCTTTTGAGC-3′
	Reverse: 5′-CTTGGAAGGACACCAGTCCC-3′
UBE2C (human)	Forward: 5′-GATGACCCTCATGGCAGTGG-3′
	Reverse: 5′-CACACAAGGGGCTTGCTAGA-3′
TP53 (human)	Forward: 5′-ACCTATGGAAACTACTTCCTGAAA-3′
	Reverse: 5′-ACCATCGCTATCTGAGCAGC-3′
GAPDH (human)	Forward: 5′-TCAGACACCATGGGGAAGGT-3′
	Reverse: 5′-TCCCGTTCTCAGCCATGTAG-3′
