# Supplementary figures and images for: Bone Marrow Mesenchymal Stem Cell-Derived Exosomes Inhibit Triple-Negative Breast Cancer Cell Stemness and Metastasis via an ALKBH5-Dependent Mechanism
Source: Cancers (Basel). 2022 Dec 9;14(24):6059. doi: 10.3390/cancers14246059 (PMC9776833; doi:10.3390/cancers14246059)

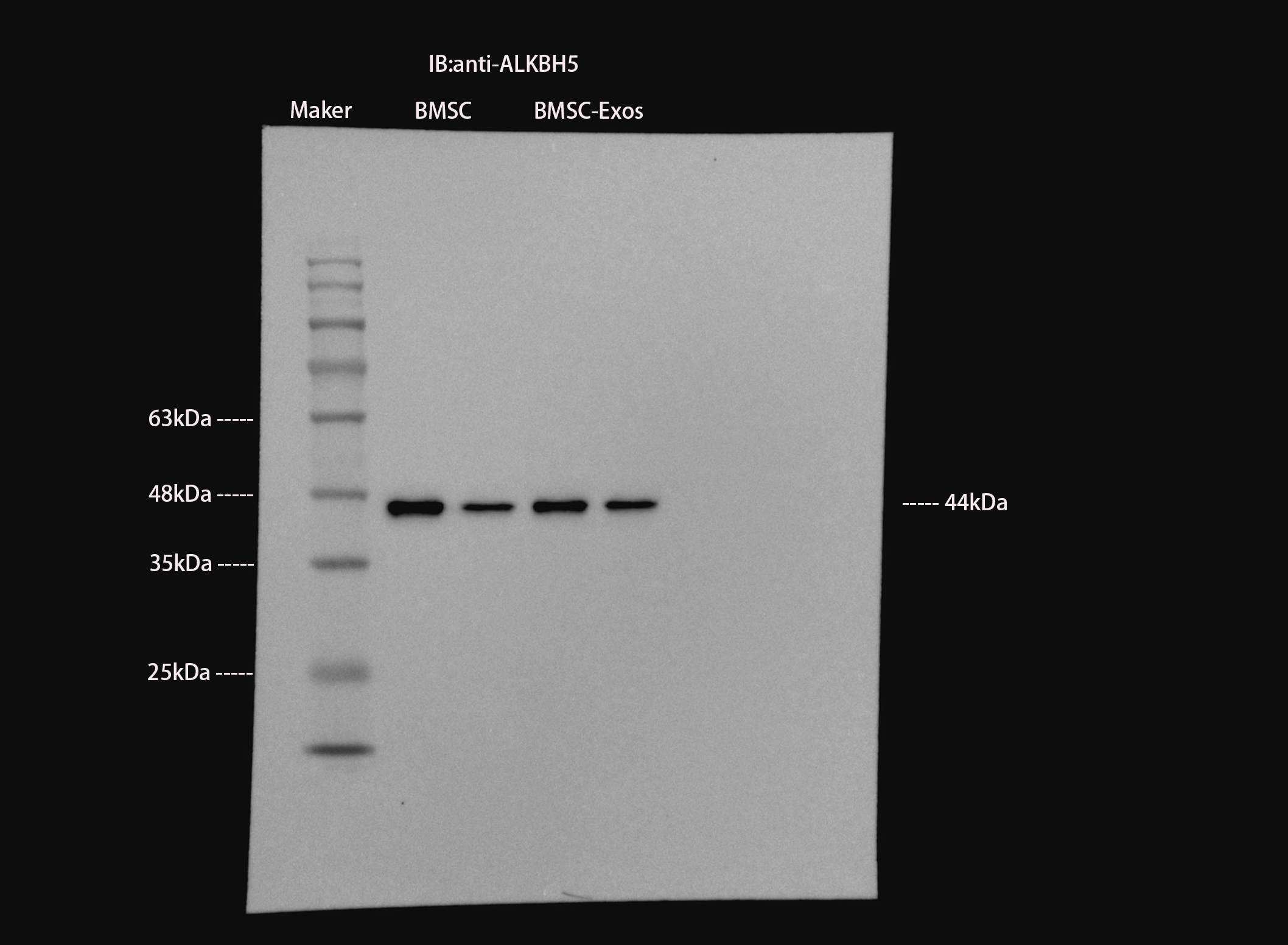

Supplement: Supplementary file 1 [file cancers-14-06059-s001.zip › Figure S1-Alkbh5.png]

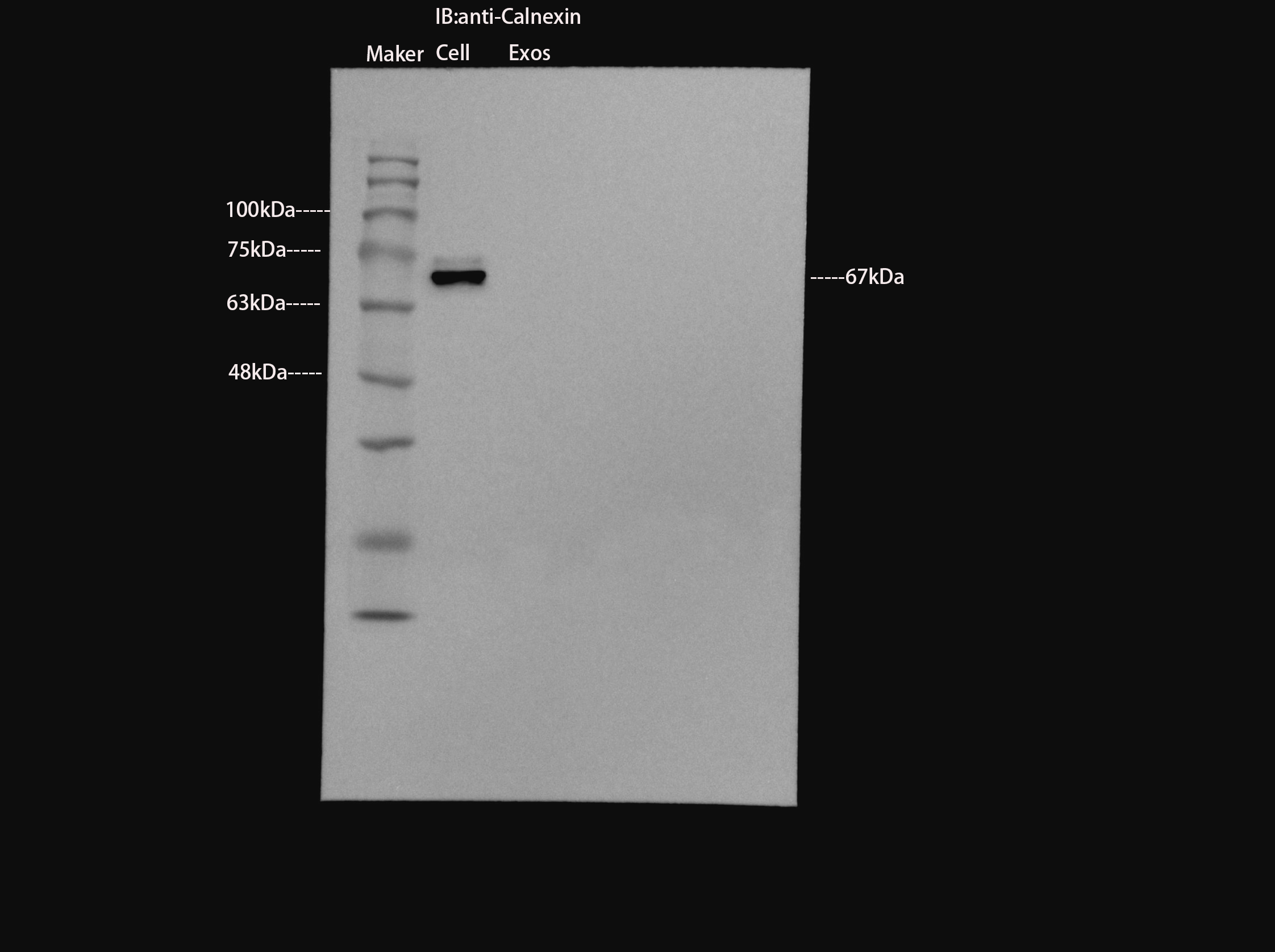

Supplement: Supplementary file 1 [file cancers-14-06059-s001.zip › Figure S1-Calnexin.png]

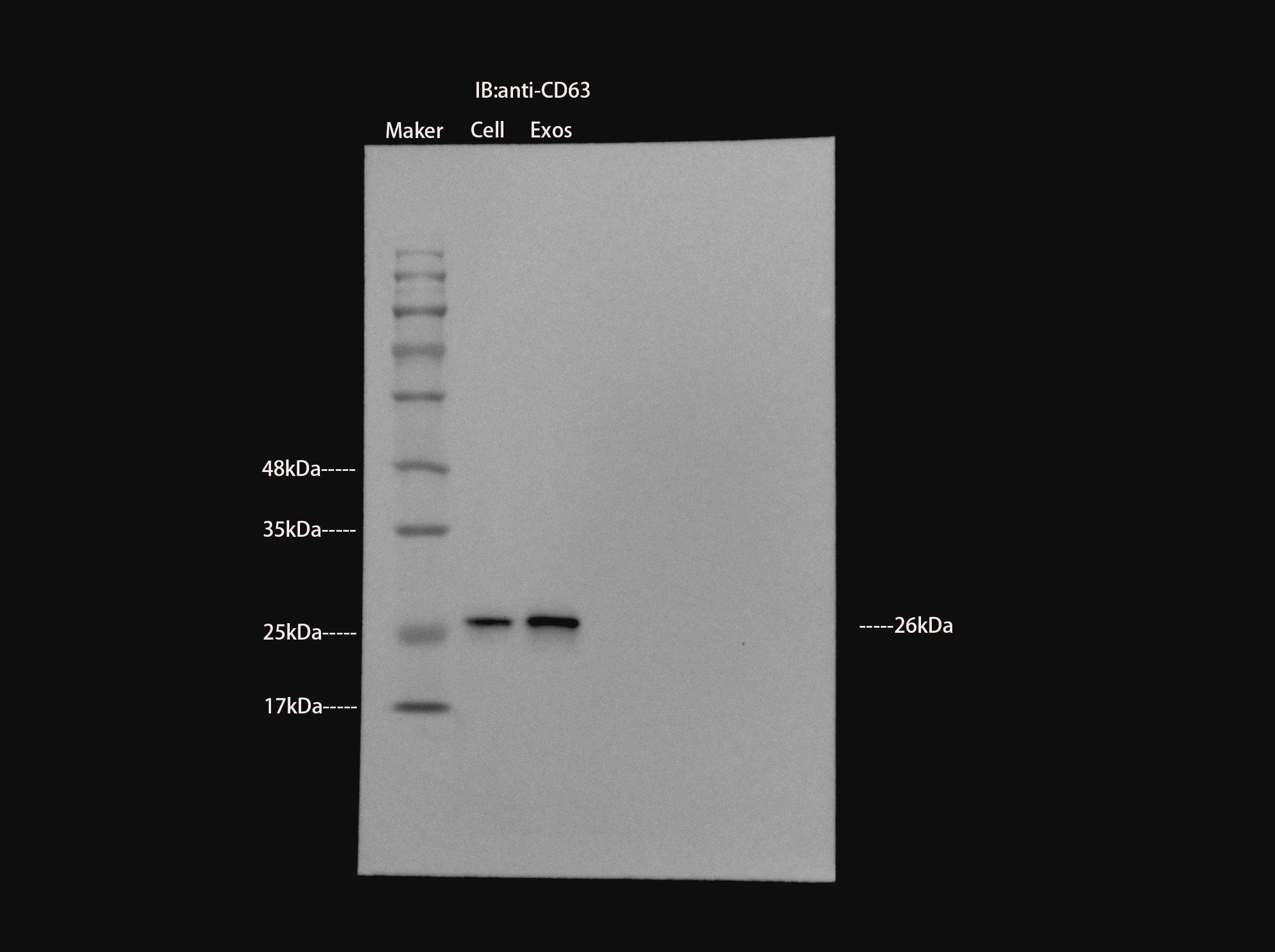

Supplement: Supplementary file 1 [file cancers-14-06059-s001.zip › Figure S1-CD63.png]

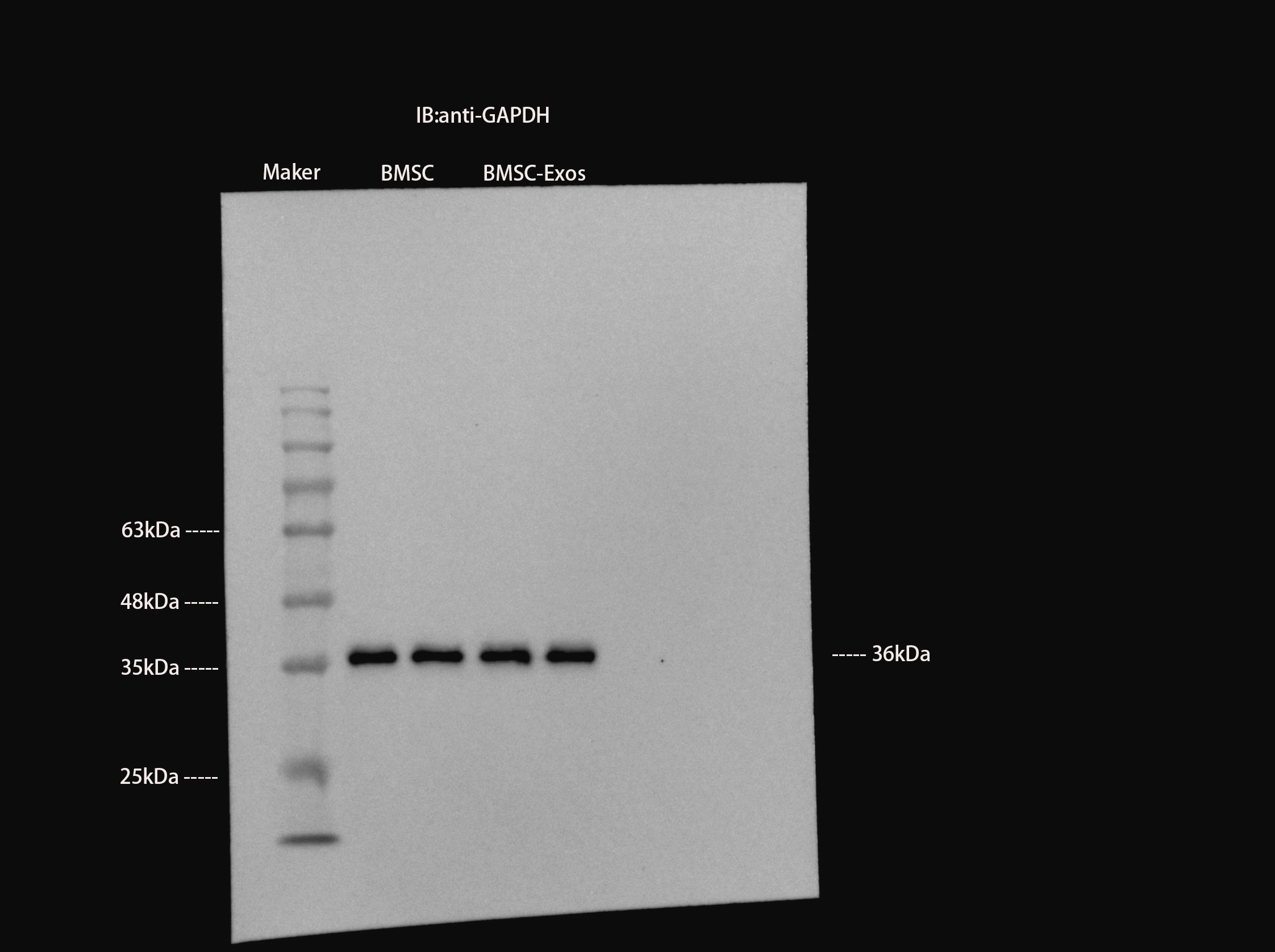

Supplement: Supplementary file 1 [file cancers-14-06059-s001.zip › Figure S1-GAPDH.png]

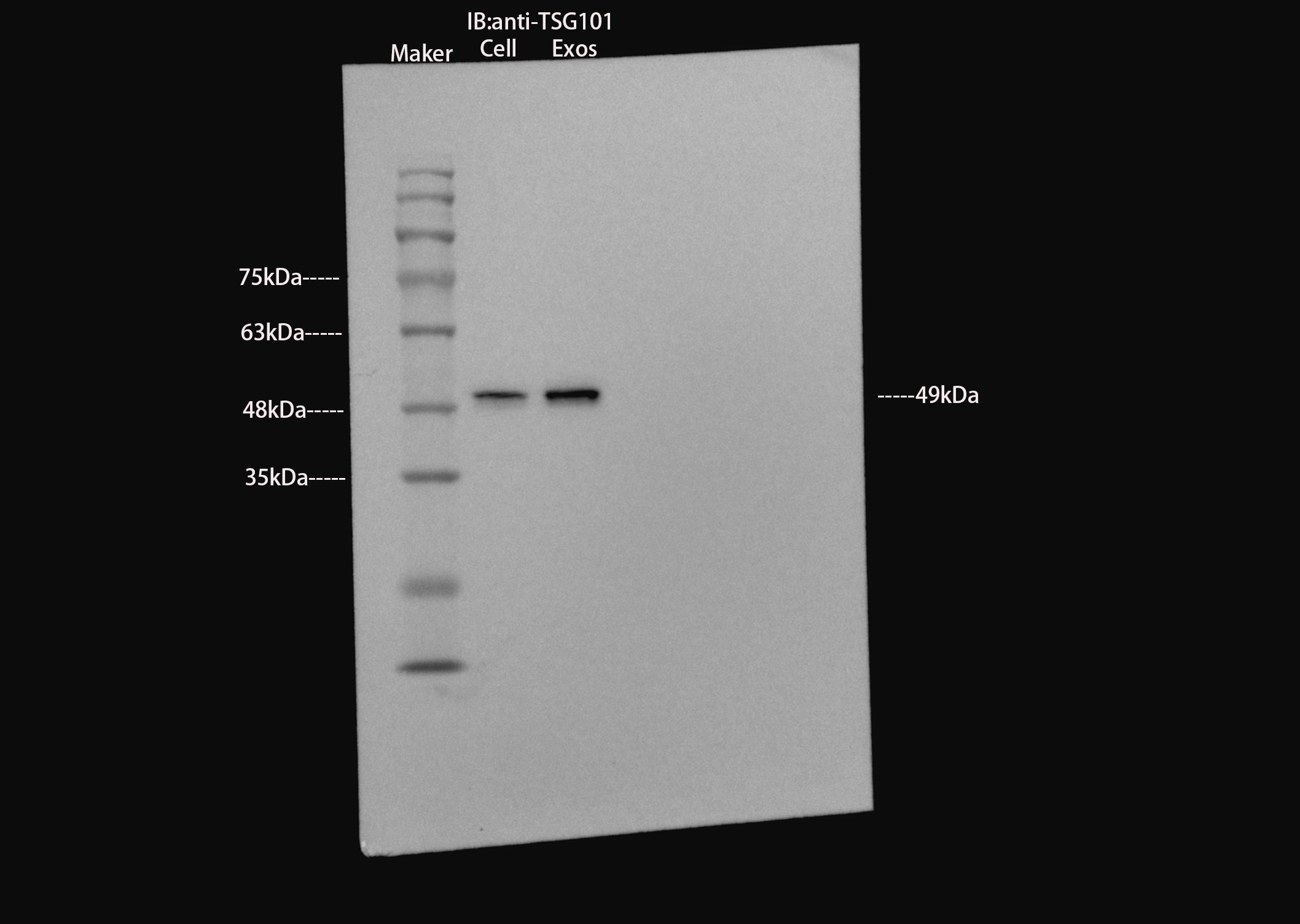

Supplement: Supplementary file 1 [file cancers-14-06059-s001.zip › Figure S1-TSG101.png]

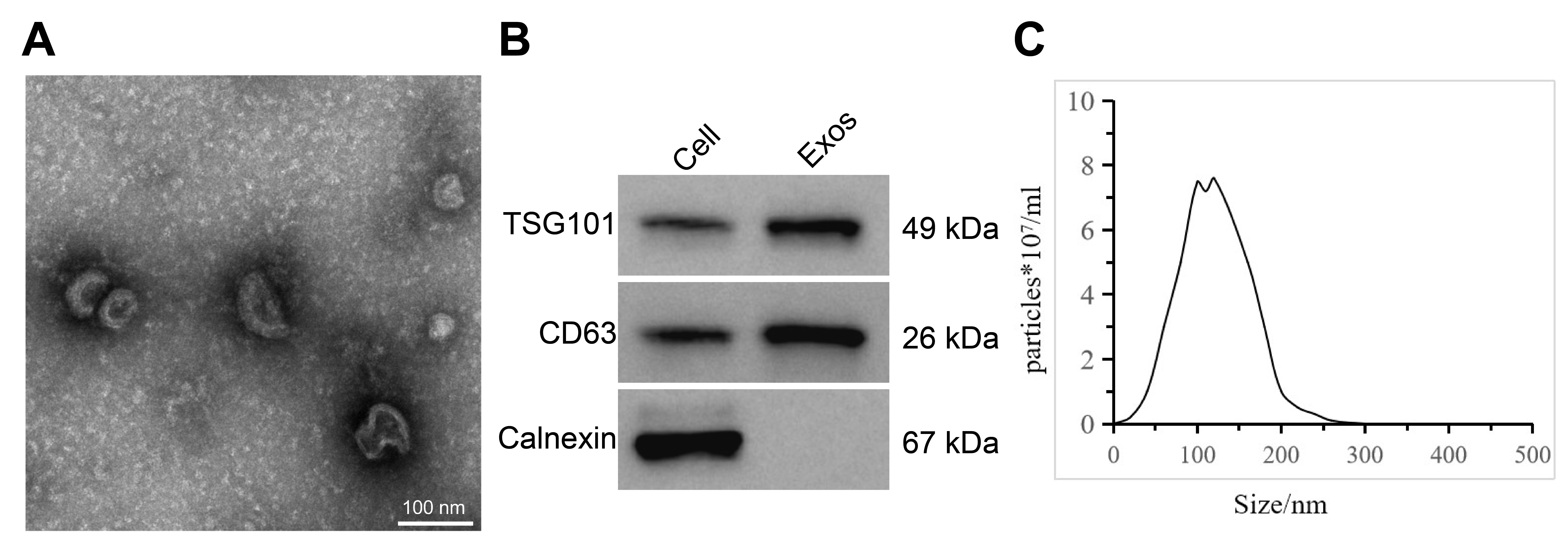

Supplement: Supplementary file 1 [file cancers-14-06059-s001.zip › Figure S2.jpg]
